# Tissue-specific molecular responses to salinomycin and their modulation by ionophore–antibiotic interactions in turkeys^☆^

**DOI:** 10.1016/j.psj.2026.107509

**Published:** 2026-07-27

**Authors:** İlksen Berfin Ekinci, Anna Sławińska, Małgorzata Olejnik

**Affiliations:** Department of Basic and Preclinical Sciences, Institute of Veterinary Medicine, Faculty of Biological and Veterinary Sciences, Nicolaus Copernicus University in Toruń, Gagarina 7, Toruń, 87-100, Poland

**Keywords:** Salinomycin, Ionophore toxicity, Drug–drug interactions, Species susceptibility, Turkey

## Abstract

Salinomycin (SAL) is a widely used ionophore with a narrow safety margin, and its interactions with co-administered drugs may modulate toxicity, especially in susceptible species such as turkeys. Yet, the molecular mechanisms underlying these interactions in turkeys are not fully understood. This study aimed to characterize tissue-specific transcriptional responses to SAL and to evaluate how co-exposure with monensin (MON), enrofloxacin (ENR), and tiamulin (TIAM) modulates these effects. Using a hypothesis-driven RT-qPCR approach based on prior transcriptomic findings, we assessed the expression of genes related to cell-cycle regulation, mitochondrial function, inflammatory signaling, and xenobiotic metabolism in turkey heart and liver tissues. SAL induced non-linear and tissue-specific transcriptional responses, and the heart was the most affected tissue. In the heart, SAL affected genes associated with stress signaling, cell-cycle, and mitochondrial function, whereas in liver, responses were limited. Co-administration with MON, ENR, and TIAM attenuated SAL-induced transcriptional responses in the heart, indicating interaction-dependent modulation. Triple co-exposure (MON+ENR+SAL) caused more pronounced suppression of transcriptional activity and extended effects to mitochondrial-related pathways in both tissues. These findings demonstrate that the heart is the primary target of SAL toxicity in turkeys. Furthermore, ionophore–antibiotic interactions significantly modulate SAL-induced molecular responses in a treatment-dependent manner. This study provides mechanistic insight into drug–drug interactions in poultry and highlights candidate biomarkers for monitoring toxicity in poultry.

## Introduction

Salinomycin (**SAL**) is a polyether ionophore widely used as an anticoccidial agent in poultry. Although considered safe at recommended doses, SAL has a narrow safety margin, and misuse or interactions with other drugs may lead to toxicity (Noack et al., 2019). Clinical signs of SAL intoxication include muscle weakness, reduced feed intake, impaired growth, and increased mortality (Holliman et al., 2011). The susceptibility to SAL toxicity varies between species. Chickens tolerate feed concentrations up to 70 mg/kg (Novilla, 2018), whereas turkeys are highly sensitive, with toxicity reported at concentrations as low as 9–24 mg/kg feed (Koutoulis et al., 2013, Potter et al., 1986, Van Assen, 2006). Therefore, turkeys are more susceptible to SAL compared to chickens.

Ionophores are often co-administered with other veterinary drugs (Islam et al., 2009; Lanckriet et al., 2010; Szűcs et al., 2004). Monensin (**MON**) is an ionophore coccidiostat routinely added to feed, and its administration with other ionophores or antibiotics can cause muscle weakness, poor growth, and increased mortality in poultry (Dowling, 1992; Gómez et al., 2025; Islam et al., 2008; Laczay et al., 1990). A recent study demonstrated that MON (120 ppm) co-exposure with tiamulin (**TIAM**) (150 ppm), a pleuromutilin antibiotic, causes reduced feed/water intake, muscle weakness, and triggers degeneration of cardiomyocytes and skeletal myocytes in brown pullets (Gómez et al., 2025). At the molecular level, Szűcs et al. (2004) found that co-exposure of MON and TIAM affects MON biotransformation by impairing cytochromes P450 (**CYP450**) enzyme activity. MON combined with sulfamethazine has caused hepatotoxicity and affected CYP450-related drug metabolism in rats (Zhao et al., 2022). Furthermore, studies demonstrated cardiomyopathy/myopathy occurs when tiamulin is combined with ionophores, and the molecular mechanisms can be explained with CYP450 inhibition (Ekinci et al., 2023). Similar to these findings, Chen et al. (2023) demonstrated impaired CYP3A4 activity in chickens after enrofloxacin (**ENR**) and SAL combined administration in a dose-dependent manner. However, most studies have investigated ionophore-drug interactions in chickens or non-avian models, and to date, there is no study in the literature that focuses on the impact of combined treatment toxicity at the molecular level in turkeys.

In our previous comparative transcriptomic study, we demonstrated that turkeys exposed to SAL (21 mg/kg) exhibited impaired cell-cycle activity in the heart and altered xenobiotic metabolism in the liver (Ekinci et al., 2025). These findings provided candidate genes and pathways that may explain the high susceptibility of turkeys; however, transcriptomic data require validation through a more targeted and sensitive approach. Reverse transcription quantitative PCR (**RT-qPCR**) is a crucial technique for analyzing the relative expression of a target gene. It provides high sensitivity and specificity for detecting mRNA expression changes in genes, especially in pathways of toxicological relevance (Joseph, 2017; Walker, 2001).

Based on our previous transcriptomic dataset, we selected genes involved in cell-cycle regulation, mitochondrial function, inflammatory signalling, and xenobiotic metabolism as potential molecular markers of SAL toxicity in turkeys. To evaluate their suitability as early, sensitive biomarkers and to characterize dose–response relationships, we exposed birds to increasing SAL concentrations in feed. In addition, because ionophores are commonly used with antibiotics, we also investigated whether SAL co-administration with MON, ENR, and TIAM modifies SAL-induced transcriptional responses. This study provides molecular mechanisms of SAL toxicity in turkeys and demonstrates potential biomarkers for monitoring ionophore drug interactions in veterinary medicine.

## Materials and methods

### Ethical approval and animal housing

Ethical approval was obtained from the Local Ethics Committee for Animal Experimentation in Bydgoszcz, Poland (Approval No. 25/2022 for the dose-effect relationships and Approval No. 19/2024 for the interaction of SAL with other xenobiotics). The study was conducted in accordance with European regulations. All procedures were performed and reported in compliance with the ARRIVE guidelines (https://arriveguidelines.org). One-day-old female turkeys obtained from a commercial hatchery were used in both experiments. Birds were reared in litter-covered floor pens measuring 3 m², at a stocking density of four birds per pen. Feed and water were provided *ad libitum* throughout the study. Animals were monitored daily, and detailed clinical examinations were performed weekly.

### Feed preparation

SAL and MON were administered through feed, whereas ENR and TIAM were administered through drinking water. Drinking water was prepared daily. Experimental feeds for the dose–effect relationship experiment and for SAL+MON treatment scheme were prepared by Zoolab (Sedziszow, Poland). Feeds used for the interaction scheme involving SAL, MON, ENR, and TIAM were prepared by Poznan University of Life Sciences (Poznan, Poland). Sacox 120 and Coxidin 200 were used as the sources of SAL and MON, respectively. To prepare SAL-containing diets, an intermediate premix containing 500 mg SAL/kg ground turkey feed was mixed with complete feed and granulated. For MON-containing diets, Coxidin 200 premix was incorporated into complete feed before granulation. The concentrations of SAL, MON, and other ionophore coccidiostats in each feed batch were checked using validated in-house LC–MS/MS and HPLC–UV methods described by Olejnik et al. (2013) and Pietruk et al. (2015). The analyzed concentrations of ionophores in the experimental feeds are shown in Table 1, Table 2. ENR and TIAM were prepared in drinking water using Enro-Sol (100 mg enrofloxacin/ml; Biowet, Poland) and Tiamogen (450 mg tiamulin hydrogen fumarate/g powder; Biofaktor, Poland), respectively.

**Table 1:** Concentrations of salinomycin and other ionophore coccidiostats in feeds used in the dose–response experiment.

Type of feed	Determined SAL^a^ concentration (mg/kg)	Determined LAS^b^, MAD^c^, MON^d^, NAR^e^, SEMD^f^ concentration (mg/kg)
Pre-experimental period	<LOD	<LOD^g^
Control group	<LOD	<LOD
0.7 mg/kg feed	0.590±0.062	<LOD
2.1 mg/kg feed	2.04±0.19	<LOD
7.0 mg/kg feed	7.70±0.67	<LOD
21 mg/kg feed	21.4±1.8	<LOD

a Salinomycin

b Lasalocid

c Maduramicin

d Monensin

e Narasin

f Semduramicin

g Limit of detection.

**Table 2:** Concentrations of salinomycin, monensin, and other ionophore coccidiostats in feeds used in the interaction experiment.

Type of feed	Determined SAL^a^ concentration (mg/kg)	Determined MON concentration (mg/kg)	Determined LAS^b^, MAD^c^, MON^d^, NAR^e^, SEMD^f^ concentration (mg/kg)
Starter Blank 0-5 w	<LOD	<LOD	<LOD^g^
Starter MON 0-5 w	<LOD	120±16.4	<LOD
Grower Blank 6-10 w	<LOD	<LOD	<LOD
Grower MON 6-10 w	<LOD	116±11.9	<LOD
Grower Blank 11-13 w	<LOD	<LOD	<LOD
Grower MON 11-13 w	<LOD	112±8.3	<LOD
Grower SAL 11-13 w	0.748±0.058	<LOD	<LOD
Grower MON+SAL 11-13 w	0.644±0.036	111±11.0	<LOD

a Salinomycin

b Lasalocid

c Maduramicin

d Monensin

e Narasin

f Semduramicin

g Limit of detection.

### Dose–effect relationship experiment

At 11 weeks of age, birds were randomly assigned to five groups: one control group without SAL and four SAL-treated groups. The SAL-treated groups received feed containing SAL at 0.7, 2.1, 7.0, or 21 mg/kg feed from week 11 to week 13.

### Interaction experiment of SAL with other xenobiotics

**Fig. 1:**
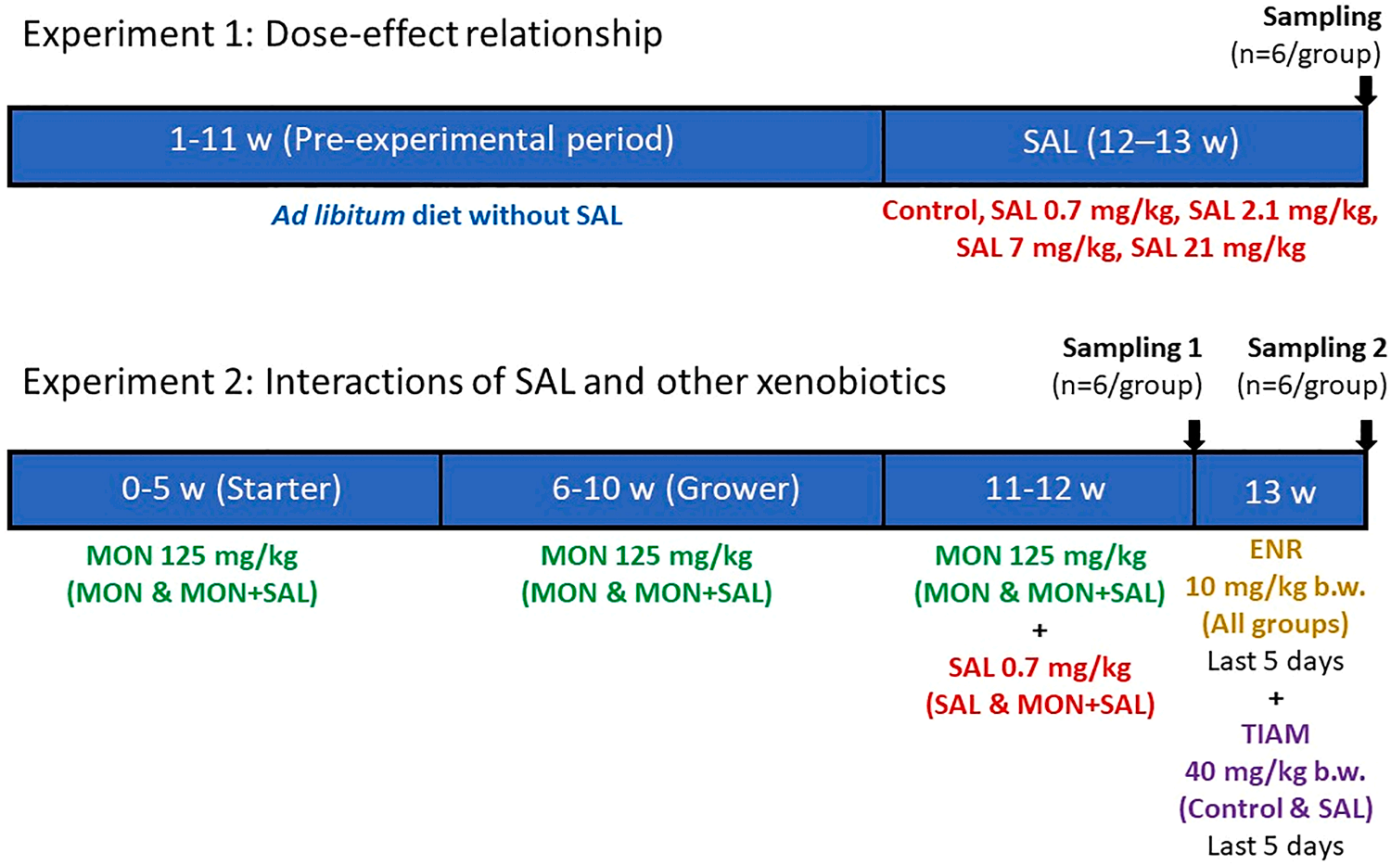
Experimental design. Experiment 1: increasing doses of salinomycin (SAL) (0, 0.7, 2.1, 7.0, and 21 mg/kg) were administered in feed during weeks 12-13, with sampling points (n = 6 per group) at the end of the experiment. Experiment 2: interactions of SAL with monensin (MON), tiamulin (TIAM), and enrofloxacin (ENR) were tested. MON (125 mg/kg) was administered in feed from day 1 to MON and MON+SAL groups. SAL (0.7 mg/kg) was administered in feed during weeks 11-12 to SAL and MON+SAL groups. Sampling 1 was performed at the end of week 12 (n = 6 per group; Control, MON, SAL, MON+SAL) before antibiotic administration. During the last 5 days of the experiment (week 13), animals received ENR (10 mg/kg b.w., all groups) or TIAM (40 mg/kg b.w., Control and SAL groups only) in drinking water. Sampling 2 was performed at the end of week 13 (n=6 per group; Control, Control+ENR, Control+TIAM, SAL+ENR, SAL+TIAM, MON+ENR, MON+SAL+ENR). w: week.

The SAL–xenobiotic interaction experiment included two treatment schemes. In the first scheme, the interaction between SAL and MON was evaluated. In the second scheme, SAL was tested in combination with MON and the antibiotics ENR and TIAM. The 0.7 mg/kg feed dose of SAL was selected based on preliminary findings identifying this concentration as the lowest observed effect level (LOEL) in turkeys. MON was administered at 125 mg/kg feed from day 1 to the MON and MON+SAL groups. Birds in the SAL and MON+SAL groups started receiving SAL-containing feed after week 10. After two weeks of SAL exposure, at the end of week 12, the first scheme included four groups: Control, MON, SAL, and MON+SAL. During the last five days of the experiment, ENR was administered at 10 mg/kg b.w., whereas TIAM was administered at 40 mg/kg b.w. Because of the known interaction between TIAM and ionophores, TIAM was not administered to MON-treated turkeys. The second scheme included seven groups: Control, Control+ENR, Control+TIAM, SAL+ENR, SAL+TIAM, MON+ENR, and MON+SAL+ENR. The overall experimental design is summarized in Fig. 1.

### Tissue collection and RNA isolation

At the end of each experiment, six birds per group were randomly selected for tissue sampling. Birds were sedated with isoflurane and euthanized by cervical dislocation. Heart and liver samples were collected immediately, divided into smaller fragments, transferred into fixRNA solution (EURx, Poland), and kept at −80°C until qPCR analysis. Total RNA was extracted from 100 mg of heart or liver tissue following the protocol described by Ekinci et al. (2025). RNA concentration and quality were measured using the QuantiFluor® RNA System kit with a Quantus™ Fluorometer (Promega, USA). RNA purity was evaluated using a NanoDrop One/OneC spectrophotometer (Thermo Fisher Scientific, USA).

### Target gene selection

In our previous transcriptomic study (Ekinci et al., 2025), we identified 4,312 differentially expressed (DE) genes in turkey heart and 3,876 DE genes in turkey liver (|log₂FC| ≥ 1, p-adj ≤ 0.05) between the SAL-exposed (21 mg/kg) and control (unexposed) groups. To understand the biological roles of DE genes, in each tissue, DE genes were subjected to pathway enrichment analysis using the Reactome. We found that the impaired cell-cycle and innate immune system in the heart, and xenobiotic metabolism in the liver. For this study, we then selected six genes from the heart and eight genes from the liver based on the pathway enrichment results. Each target gene significantly changed between the SAL-exposed and control groups and had a role in cardiac and/or hepatic physiology in avian or mammalian models. Genes with moderate log₂FC values were also included due to their key roles in pathways associated with SAL toxicity. The selected genes and their tissue-specific roles, corresponding log₂FC values and associated Reactome pathways are summarized in Table 3. Detailed transcriptomic data including adjusted p-values, and supporting references are provided in Supplementary File 1.

**Table 3:** List of target genes selected for RT-qPCR.

Gene Symbol	Gene Name	Tissue	Role in Tissue	Log_2_FC	Reactome Pathway
*RBL1*	RB Transcriptional Corepressor Like 1	Heart	Cell cycle regulator	-1.05	Cell Cycle
*CDKN3*	Cyclin-dependent kinase inhibitor 3		Cyclin inhibitor	-2.09	Cell Cycle Checkpoints
*BRCA2*	Breast cancer type 2		Maintains genomic stability	-1.52	Cell Cycle
*NFKBIA*	NFKB Inhibitor Alpha		Suppresses NFkb pathway activation	0.52	Innate Immune System
*COX10*	Cytochrome C Oxidase Assembly Factor Heme A:Farnesyltransferase		Component of the electron transport chain	0.45	Respiratory electron transport
*LCN2*	Lipocalin 2		Innate/stress response marker	1.55	Innate Immune System
*PLCB2*	Phospholipase C Beta 2	Liver	Second messenger regulator	3.81	GPCR downstream signalling
*CAMK2G*	Calcium/Calmodulin Dependent Protein Kinase II Gamma		Regulator of calcium ion transport	1.61	G alpha (i) signalling events
*NFATC2*	Nuclear Factor Of Activated T Cells 2		Induce T cell activation via cytokines	3.61	Signal Transduction
*CREB1*	CAMP Responsive Element Binding Protein 1		Regulates cellular response	2.80	GPCR downstream signalling
*CYP21A2*	Cytochrome P450 Family 21 Subfamily A Member 2		Steroid/xenobiotic metabolism	-3.53	Translation
*DNM1L*	Dynamin 1 Like		Regulator for mitochondria membrane fission	-0.48	Translation
*MFF*	Mitochondrial Fission Factor		Participates in mitochondria division	-1.21	Translation
*MFN2*	Mitofusin 2		Participates in mitochondrial fusion	1.09	Translation

Genes were selected from the previous transcriptomic study (Ekinci et al., 2025). Log₂FC values represent differential gene expression between SAL-exposed (21 mg/kg) and control groups. Positive log₂FC values indicate upregulation, negative values indicate downregulation.

### Reverse Transcription-quantitative PCR (RT-qPCR)

Primer pairs for target and reference genes were designed using Primer-BLAST (Ye et al., 2012), and their sequences are provided in Table 4. For each sample, 2.5 μg of total RNA was used for reverse transcription with the Maxima First Strand cDNA Synthesis Kit for RT-qPCR (Thermo Fisher Scientific, USA) according to the manufacturer’s protocol. The synthesized cDNA was diluted to 100 ng/μL and stored at −20°C until qPCR analysis. Real-time PCR was performed using Maxima SYBR Green qPCR Master Mix (Thermo Fisher Scientific, USA) on a LightCycler 480 II instrument (Roche Diagnostics, Switzerland). Each 10 μL reaction contained 2 μL of diluted cDNA, 1 μM forward primer, and 1 μM reverse primer. Target genes were analyzed in six biological replicates, with two technical replicates per sample. Reference genes were analyzed using three technical replicates. The amplification protocol included an initial denaturation step at 95°C for 15 min, followed by 40 cycles of 95°C for 15 s, 58°C for 15 s, and 72°C for 30 s. Melting-curve analysis was performed after amplification to confirm the specificity of PCR products. Reference genes were selected based on their common use (Brady et al., 2023) and then evaluated using RefFinder (Xie et al., 2023). According to the RefFinder ranking, phosphoglycerate kinase 1 (*PGK1*) and succinate dehydrogenase complex flavoprotein subunit A (*SDHA*) showed the highest stability in turkey heart and liver based on BestKeeper values. Therefore, target gene expression was normalized using the average cycle threshold (Ct) values of *PGK1* and *SDHA*. Relative mRNA expression between experimental groups was calculated using the 2^−ΔΔCt^ method. Primer efficiency was assessed from standard curves generated using five-point, 10-fold serial cDNA dilutions performed in duplicate reactions. Efficiency was calculated using E% = (10−1/slope − 1) × 100, and primer pairs were accepted when efficiency ranged from 90% to 110% with R² ≥ 0.98 (Supplementary File 2).

**Table 4:** List of primers for RT-qPCR used in this study (F: Forward; R: Reverse).

Gene symbol	Accession number	Primer sequences (5′ - 3′)	Size (bp)	Reference
*RBL1*	XM_010722225.3	F: CTTCAACTTCTGCTGGACCCT R: GGAAGTCTGCTGGTAAGCCTTTA	185	This study
*CDKN3*	XM_003206826.4	F: CCTGCTGCACAATTCTGGAA R: ATTGCTTGCTGAGGTGTCAC	151	This study
*BRCA2*	XM_031552053.1	F: ATGCCACCTTTTTCCTCTCCTG R: ATTCCTAAAAATAGCTGGACTCGC	180	This study
*NFKBIA*	XM_010711684.2	F: GCCACCAACTACAATGGACATAC R: TGCTCCTAAGGACAGCAGGT	90	This study
*COX10*	XM_003211410.4	F: GGAAGTCTTGATGCTGGTGCT R: GTCCAATTAAGGCCACGCAG	187	This study
*LCN2*	XM_003211161.4	F: GCCTCCAACTCCAACTGGT R: TCCGGATATAGAGGCTGTTCC	151	This study
*PLCB2*	NM_001303201.1	F: ACGTATCAGAACAAGGAGATGGA R: ATGGAAAGTGAGGTCCACCA	192	This study
*CAMK2G*	XM_019617944.2	F: CACCAGAAACTGGAACGTGA R: TGATGAATACAGTGGCTGGC	194	This study
*NFATC2*	XM_031556555.1	F: GCTCATGAACTTCCGATGGT R: TCTACAAAGAGCATGCTAGGC	203	This study
*CREB1*	XM_010713479.3	F: CATCTTCTGCACCCACGG R: GAGATCTGAACTGTCTGGACC	127	This study
*CYP21A2*	XM_019610239.2	F: AGCTATATCGGGGCTCTCTG R: CGCCAGTTCTCATATTGCTCA	131	This study
*DNM1L*	XM_031556048.1	F: ATGAGTTCCTACCCGAGCG R: ATCGCTGTAGGATGTACCCC	144	This study
*MFF*	XM_019611616.2	F: CCAGGAATGACAAAGGTGCCT R: GGATCCACTTCCCGTGCAATT	173	This study
*MFN2*	XM_010716427.3	F: CCCCTCAAAATGAAGAGGTTCG R: CCTGTTGCACTGATGGTGTC	204	This study
*PGK1*	100550807	F: CAAAGGCCCTTGAGAGTCCA R: ATGCCATTCCACCACCAATG	132	Brady et al., 2023
*SDHA*	100546340	F: CTAAGCCACTCCAAGGCCAA R: ATAGGAGCGAATAGCAGGCG	173	Brady et al., 2023

### Statistical analysis

The adequacy of the experimental sample size was evaluated using G*Power 3.1 software (Faul et al., 2009). A sample size of six birds per group was considered sufficient based on the expected effect size and a statistical power of 0.80 (1−β). Relative mRNA expression values were first calculated in Microsoft Excel for each target gene and tissue. For each gene, normality was assessed with delta Ct values using the Shapiro-Wilk test. Because RT-qPCR expression data did not consistently meet the assumptions of normal distribution, statistical differences among groups were analyzed using the Kruskal–Wallis test. Post-hoc comparisons were performed using Dunn’s multiple comparisons test. The statistical analysis of RT-qPCR data was performed with GraphPad Prism 8.4.2 (www.graphpad.com), and P < 0.05 was considered statistically significant. No animals, experimental units, or data points were excluded from the analysis. Blinding was not performed during the experiment or data analysis.

## Results

### Salinomycin modulates cell-cycle, mitochondrial, and inflammatory responses in the heart

**Fig. 2:**
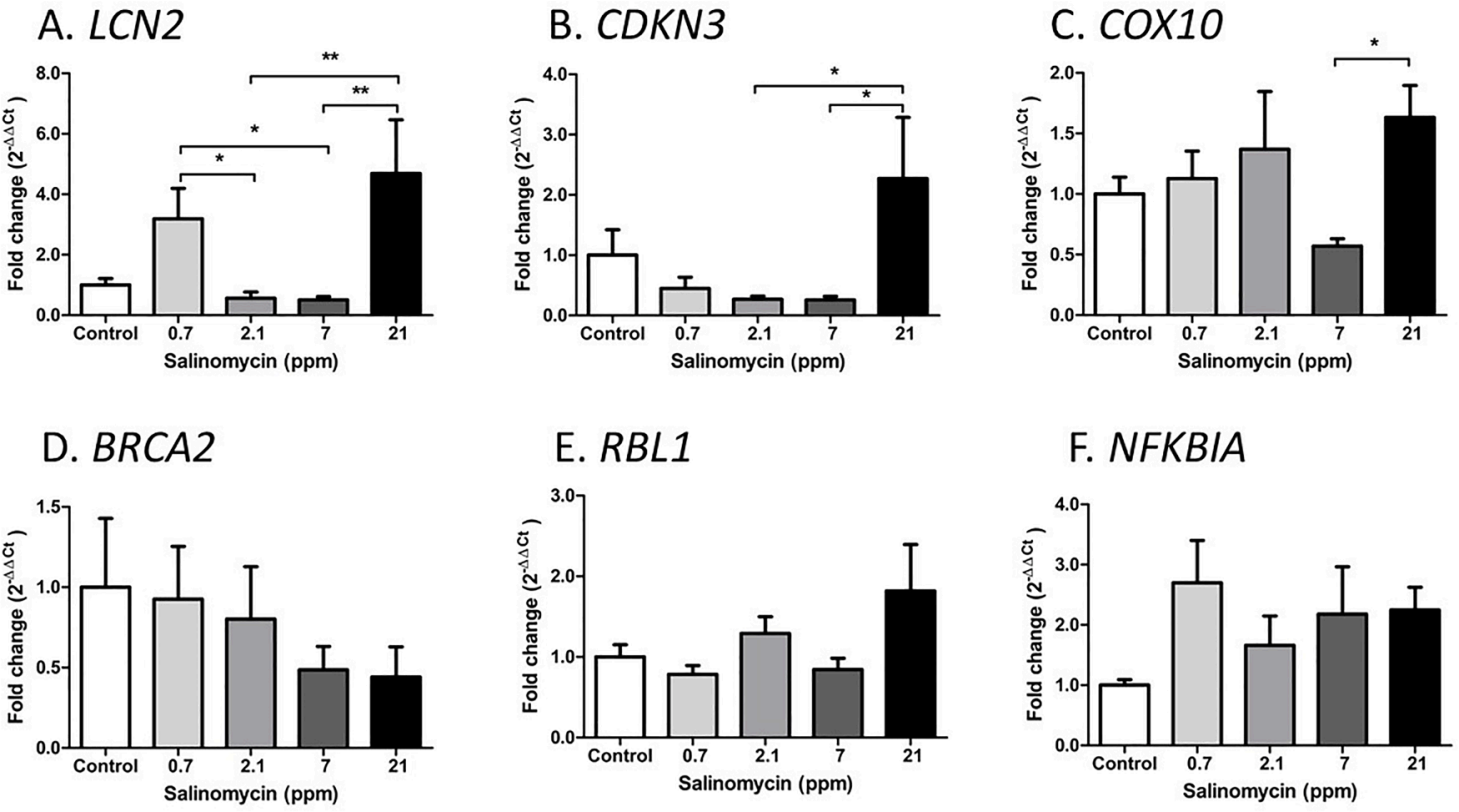
Transcriptional responses of target genes to increasing SAL doses in the heart with RT-qPCR analysis. (A) *LCN2*, (B) *CDKN3*, (C) *COX10*, (D) *BRCA2*, (E) *RBL1*, and (F) *NFKBIA*. The relative expression of each gene was calculated by the 2^−ΔΔCt^ method and normalized to the control group (=1). Dose–response between the control group and SAL doses (0.7, 2.1, 7, 21 ppm) was analyzed by Kruskal–Wallis followed by Dunn’s post-hoc test. RT-qPCR data are presented as means ± SEM (n = 6). The X-axis represents experimental groups, and the Y-axis represents the relative expression of a target gene (2^−ΔΔCt^). *P < 0.05; **P < 0.01. SAL: Salinomycin.

We performed RT-qPCR analysis to evaluate the relative mRNA expression of six target genes in turkey heart following exposure to increasing doses of SAL (0, 0.7, 2.1, 7, and 21 ppm) (Fig. 2). Among the analyzed genes, *LCN2, CDKN3*, and *COX10* showed significant differences between experimental groups (P < 0.05), whereas *RBL1, BRCA2*, and *NFKBIA* were not statistically significant (P > 0.05). *LCN2* expression was significantly higher at 0.7 ppm (3.19 fold) compared to 2.1 ppm (0.56 fold), and its expression at 21 ppm (4.68 fold) was significantly higher compared to both 2.1 ppm (0.56 fold) and 7 ppm (0.50 fold) (Fig. 2A). Similarly, *CDKN3* expression was significantly increased at 21 ppm (2.27 fold) compared to 2.1 ppm (0.26 fold) and 7 ppm (0.25 fold) (Fig. 2B). For *COX10*, expression at 21 ppm (1.63 fold) was significantly higher compared to 7 ppm (0.56 fold) (Fig. 2C).

### Salinomycin alters mitochondrial-related gene expression in the liver

**Fig. 3:**
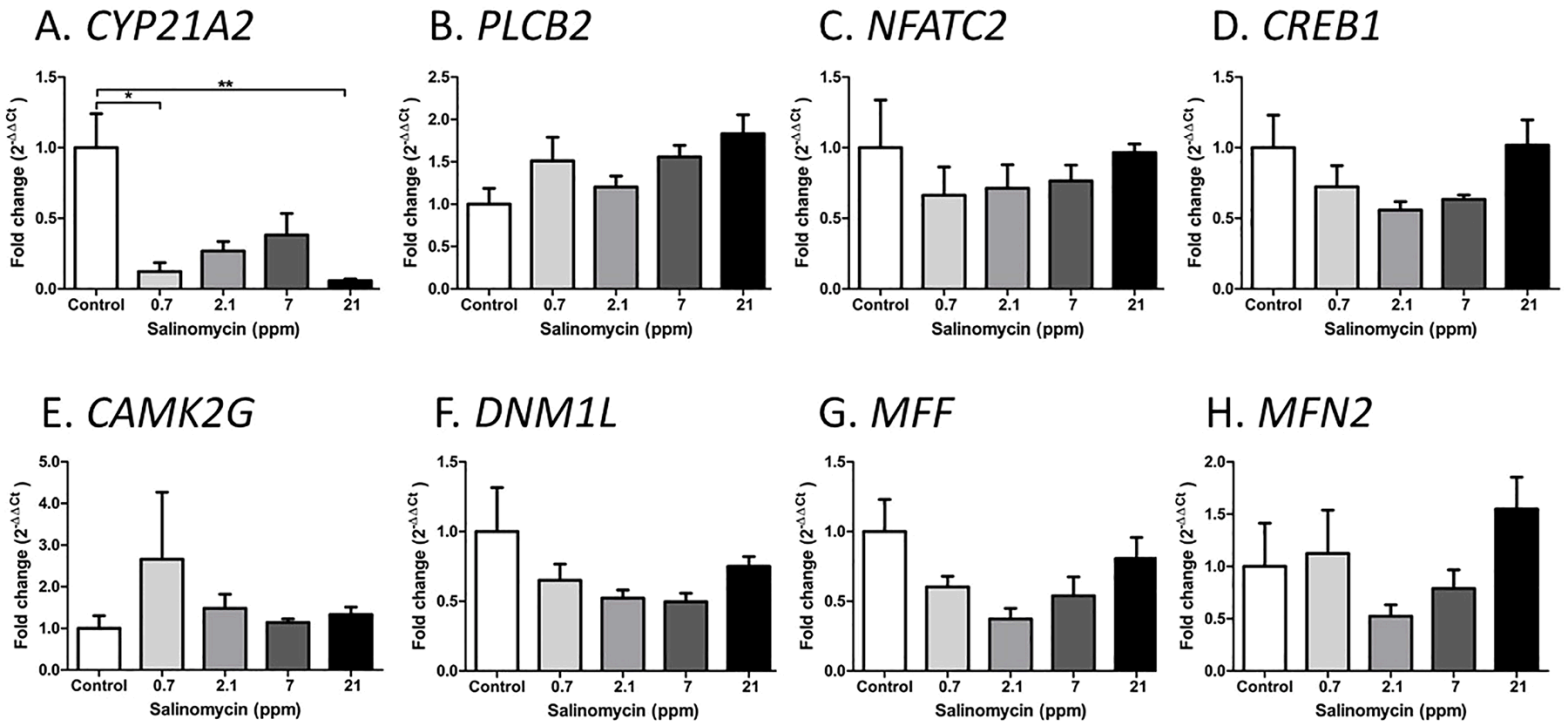
Trancriptional responses of target genes to increasing SAL doses in the liver with RT-qPCR analysis. (A) *CYP21A2*, (B) *PLCB2*, (C) *NFATC2*, (D) *CREB1*, (E) *CAMK2G*, (F) *DNM1L*, (G) *MFF* and (H) *MFN2*. The relative expression of each gene was calculated by the 2^−ΔΔCt^ method and normalized to the control group (=1). Dose–response between the control group and SAL doses (0.7, 2.1, 7, 21 ppm) was analyzed by Kruskal–Wallis followed by Dunn’s post-hoc test. RT-qPCR data are presented as means ± SEM (n = 6). The X-axis represents experimental groups, and the Y-axis represents the relative expression of a target gene (2^−ΔΔCt^). *P < 0.05; **P < 0.01. SAL: Salinomycin.

In turkey liver, the relative mRNA expression of eight target genes was analyzed following exposure to increasing doses of salinomycin (SAL) (0, 0.7, 2.1, 7, and 21 ppm) (Fig. 3). Among the analyzed genes, only *CYP21A2* showed significant differences between experimental groups (P < 0.05), whereas *PLCB2, NFATC2, CREB1, CAMK2G, DNM1L, MFF*, and *MFN2* were not statistically significant (P > 0.05). *CYP21A2* expression was significantly decreased at 0.7 ppm (0.12 fold) compared to the control group (1.0 fold). In addition, *CYP21A2* expression at 21 ppm (0.05 fold) was significantly lower compared to the control group (Fig. 3A). Taken together, the dose-associated transcriptional responses in the heart and liver were non-linear and gene-specific, with no consistent increase or decrease in expression across the tested SAL doses.

### Salinomycin and monensin co-exposure alters gene expression in heart, with no significant response in liver

**Fig. 4:**
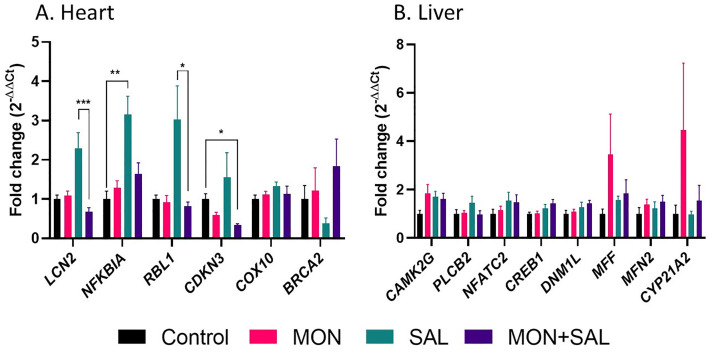
Transcriptional responses of target genes exposed to salinomycin (SAL), monensin (MON) and their combined administration (MON+SAL) in the heart (A) and the liver (B) with RT-qPCR analysis. Relative expression of each gene was calculated by the 2^−ΔΔCt^ method and normalized to the control group (=1). Statistical significance was determined using the Kruskal–Wallis test followed by Dunn’s multiple comparisons test. Data are presented as means ± SEM (n=6). The X-axis represents target genes, and the Y-axis represents the fold change of a target gene (2^−ΔΔCt^). *P < 0.05; **P < 0.01.

To assess whether co-administration of monensin (MON) modifies SAL-induced transcriptional responses, turkeys were exposed to SAL (0.7 mg/kg), MON (125 mg/kg), or their combination (MON+SAL), and the expression of target genes was analyzed in the heart and liver by RT-qPCR (Fig. 4).

The most pronounced transcriptional responses were observed in the heart, whereas no statistically significant changes were detected in the liver under the tested conditions (Fig. 4B). In the heart, *LCN2, NFKBIA, RBL1*, and *CDKN3* showed significant differences between experimental groups (P < 0.05), whereas *COX10* and *BRCA2* were not statistically significant (P > 0.05). *LCN2* expression was significantly higher in the SAL group (2.29 fold) compared to the MON+SAL group (0.68 fold). Similarly, *RBL1* expression was significantly increased in the SAL group (3.02 fold) compared to MON+SAL (0.81 fold) (Fig. 4A). For *NFKBIA*, SAL exposure significantly increased its expression (3.15 fold) compared to the control group. In contrast, *CDKN3* expression was significantly decreased in the MON+SAL group (0.34 fold) compared to control (Fig. 4A). MON alone did not significantly affect the expression of any target gene compared to control (P > 0.05). No other pairwise comparisons were statistically significant.

### Salinomycin and enrofloxacin co-exposure alters gene expression in heart, with no significant response in liver

**Fig. 5:**
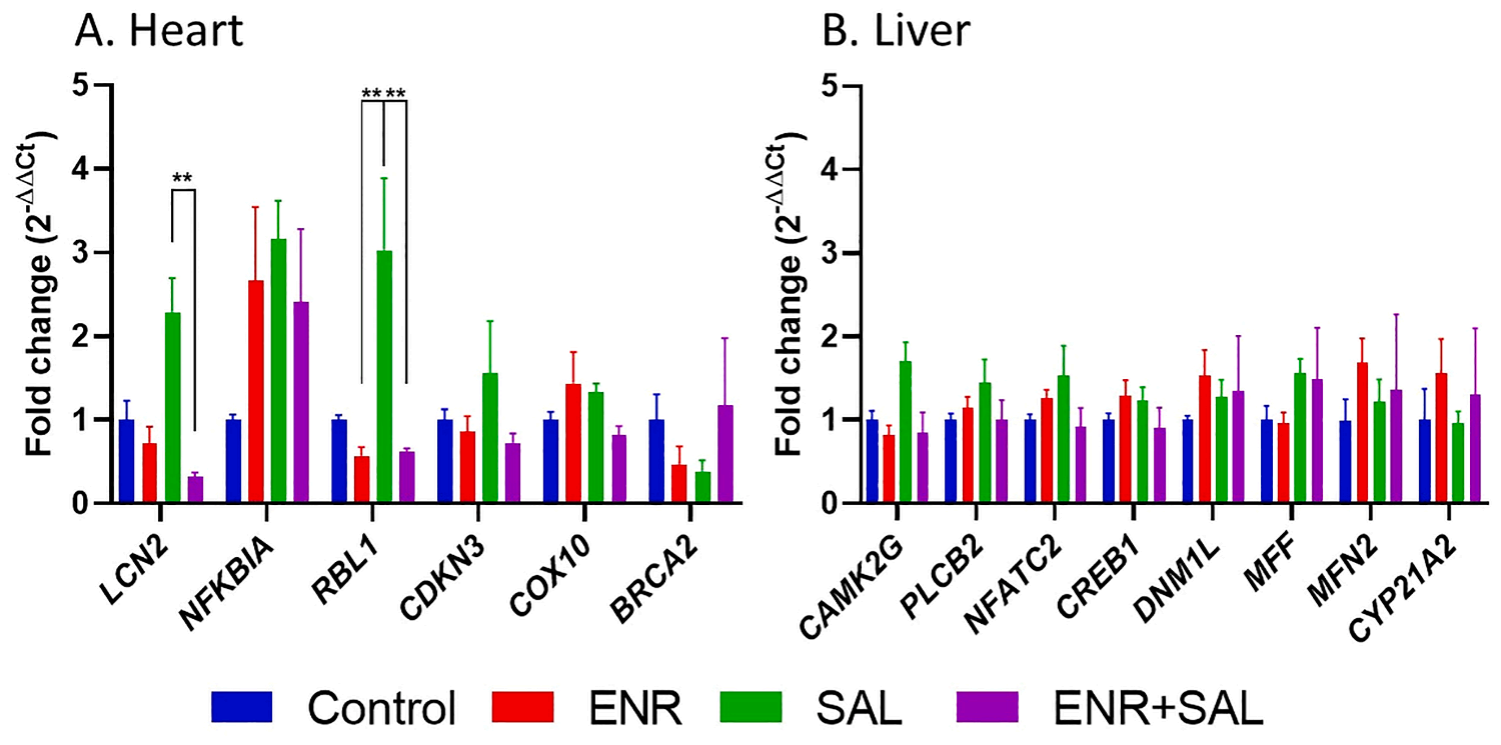
Transcriptional responses of target genes exposed to salinomycin (SAL), enrofloxacin (ENR) and their combined administration (ENR+SAL) in the heart (A) and the liver (B) with RT-qPCR analysis. Relative expression of each gene was calculated by the 2^−ΔΔCt^ method and normalized to the control group (=1). Statistical significance was determined using the Kruskal–Wallis test followed by Dunn’s multiple comparisons test. Data are presented as means ± SEM (n=6). The X-axis represents target genes, and the Y-axis represents the fold change of a target gene (2^−ΔΔCt^). *P < 0.05; ** P < 0.01.

We investigated whether antibiotic administration modifies transcriptional responses associated with SAL-induced toxicity. For this purpose, enrofloxacin (ENR) was co-administered (10 mg/kg b.w.) with SAL (0.7 mg/kg) in turkeys, and the expression of target genes was analyzed in the heart and liver by RT-qPCR. Similar to MON co-exposure (Fig. 4), the most pronounced molecular responses were observed in the heart (Fig. 5A), whereas no statistically significant changes were detected in the liver under the tested conditions (Fig. 5B). In the heart, among the six target genes, only *LCN2* and *RBL1* showed significant differences between experimental groups (P < 0.05), while the remaining genes were not statistically significant (P > 0.05). *LCN2* expression was significantly higher in the SAL group (2.29 fold) compared to the ENR+SAL group (0.32 fold) (Fig. 5A). A similar pattern was observed for *RBL1*, which was significantly upregulated in the SAL group (3.02 fold) compared to ENR+SAL (0.62 fold). In addition, *RBL1* expression was also significantly higher in the SAL group (3.02 fold) compared to the ENR-alone (0.56 fold) (Fig. 5A). ENR alone did not significantly alter the expression of any target gene compared to the control group (P > 0.05). No other pairwise comparisons were statistically significant.

### Co-exposure with monensin and enrofloxacin alters salinomycin-induced transcriptional responses in the heart and liver

**Fig. 6:**
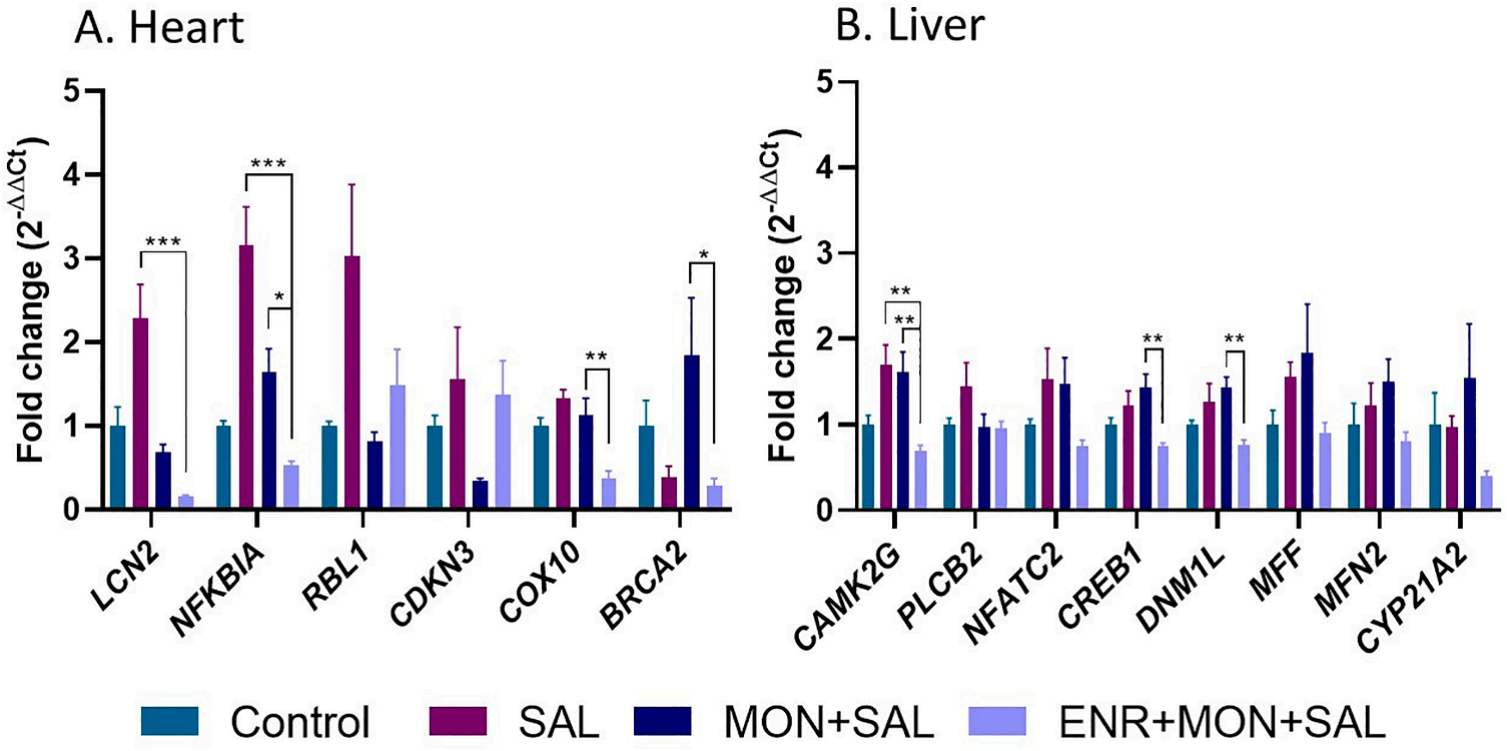
Transcriptional responses of target genes exposed to salinomycin (SAL), monensin and salinomycin (MON+SAL), and triple co-exposure to enrofloxacin, monensin and salinomycin (ENR+MON+SAL) in the heart (A) and the liver (B) with RT-qPCR analysis. Relative expression of each gene was calculated by the 2^−ΔΔCt^ method and normalized to the control group (=1). Statistical significance was determined using the Kruskal–Wallis test followed by Dunn’s multiple comparisons test. Data are presented as means ± SEM (n=6). The X-axis represents target genes, and the Y-axis represents the fold change of a target gene (2^−ΔΔCt^). *P < 0.05; **P < 0.01.

To further evaluate the combined effects of ionophore and antibiotic co-exposure on SAL-induced responses, the expression of target genes was analyzed in turkeys exposed to SAL, MON+SAL, and ENR+MON+SAL (triple co-exposure) in the heart and liver by RT-qPCR (Fig. 6). Similar to previous findings, the most pronounced transcriptional responses were observed in the heart (Fig. 6A), whereas the liver showed limited and gene-specific changes (Fig. 6B). In the heart, *LCN2, NFKBIA, COX10*, and *BRCA2* showed significant differences between experimental groups. *CDKN3* was not statistically significant (P > 0.05).

*LCN2* expression was significantly increased in the SAL group (2.29 fold) compared to the ENR+MON+SAL group (0.15 fold). A similar pattern was observed for *NFKBIA*, with higher expression in the SAL group (3.15 fold) than in the ENR+MON+SAL group (0.52 fold). In addition, *NFKBIA* expression was also higher in the MON+SAL group (1.64 fold) than in the ENR+MON+SAL group (0.52 fold). *COX10* expression was significantly altered between groups, with reduced expression in the ENR+MON+SAL group (0.37 fold) compared to the MON+SAL group (1.12 fold). For *BRCA2*, expression levels also differed significantly between groups, with higher expression in the MON+SAL group (1.84 fold) than in the ENR+MON+SAL group (0.28 fold) (Fig. 6A).

In the liver, among the eight target genes, *CAMK2G, CREB1*, and *DNM1L* showed significant differences between groups, whereas the remaining genes were not statistically significant (p>0.05). *CAMK2G* expression was significantly increased in the SAL (1.69 fold) and MON+SAL (1.61 fold) groups compared to the ENR+MON+SAL group (0.69 fold). Similarly, *CREB1* (1.43 fold) and *DNM1L* (1.27 fold) expression levels were higher in the MON+SAL group than in the ENR+MON+SAL group (*CREB1*: 0.74 fold; *DNM1L*: 0.27 fold) (Fig. 6B). No other pairwise comparisons were statistically significant.

### Tiamulin co-administration modulates salinomycin-induced transcriptional responses in the heart, with no significant effects in the liver

**Fig. 7:**
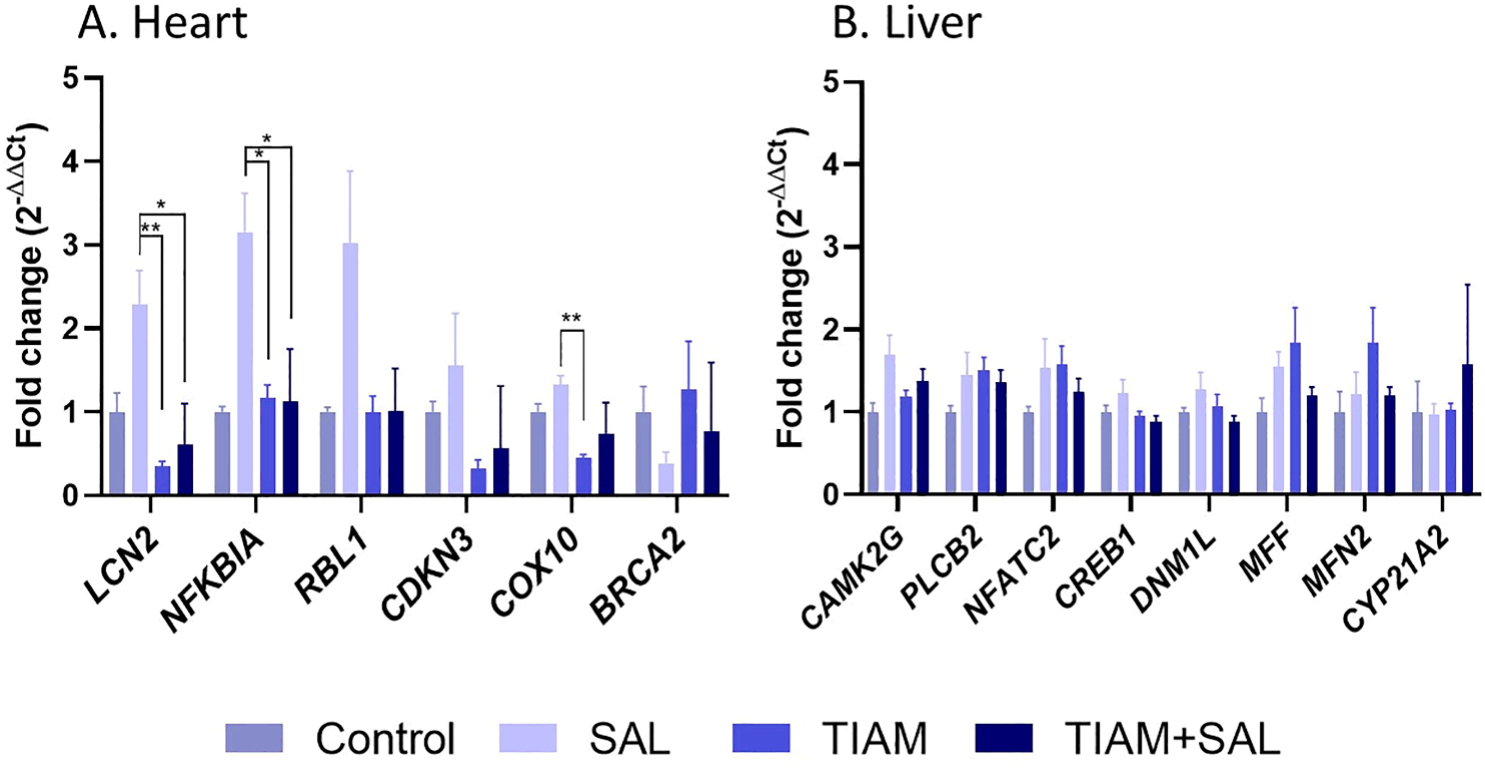
Transcriptional responses of target genes exposed to salinomycin (SAL), tiamulin (TIAM) and tiamulin and salinomycin (TIAM+SAL) in the heart (A) and the liver (B) with RT-qPCR analysis. Relative expression of each gene was calculated by the 2^−ΔΔCt^ method and normalized to the control group (=1). Statistical significance was determined using the Kruskal–Wallis test followed by Dunn’s multiple comparisons test. Data are presented as means ± SEM (n=6). The X-axis represents target genes, and the Y-axis represents the fold change of a target gene (2^−ΔΔCt^). *P < 0.05; **P < 0.01.

Next, we analyzed whether tiamulin (TIAM) co-administration modifies SAL-induced transcriptional responses. Turkeys were exposed to SAL (0.7 mg/kg), TIAM (40 mg/kg b.w.), or their combination (TIAM+SAL), and the expression of target genes was analyzed in the heart and liver by RT-qPCR (Fig. 7). Similar to previous co-exposure results, the most pronounced transcriptional responses were observed in the heart (Fig. 7A), whereas no statistically significant changes were detected in the liver under the tested conditions (Fig. 7B).

In the heart, *LCN2, NFKBIA*, and *COX10* showed significant differences between experimental groups, whereas *RBL1, CDKN3*, and *BRCA2* were not statistically significant (P > 0.05). *LCN2* expression was significantly higher in the SAL group (2.29 fold) compared to both the TIAM (0.35 fold) and TIAM+SAL groups (0.60 fold) (Fig. 7A). Similarly, *NFKBIA* expression was significantly increased in the SAL group (3.15 fold) compared to the TIAM (1.16 fold) and TIAM+SAL groups (1.12 fold) (Fig. 7A). For *COX10*, expression was significantly higher in the SAL group (1.32 fold) compared to the TIAM group (0.44 fold) (Fig. 7A). No other pairwise comparisons were statistically significant. TIAM alone did not significantly affect the expression of any target gene compared to control (P > 0.05).

## Discussion

The present study aimed to characterize the molecular signatures of SAL toxicity in turkeys and to determine how co-administration with commonly used antibiotics and ionophores modulates these responses. To address this, we evaluated dose-dependent responses of SAL and its interactions with MON, ENR, and TIAM using targeted RT-qPCR analysis in heart and liver tissues.

First, we analyzed dose-dependent response of SAL in turkeys. In the heart, the significantly changed genes were associated with stress signalling, cell-cycle regulation, and mitochondrial activity. *LCN2* demonstrated a biphasic-like response, with increased expression at both lowest and highest doses. *LCN2* encodes lipocalin-2, a multifunctional protein plays a role in inflammation, and cellular stress responses (Xu et al., 2012). Given its role, this non-linear expression pattern may reflect early adaptive responses at low doses and exacerbated damage at higher doses. *CDKN3* expression was significantly increased only at the highest SAL dose, which may indicate a high-dose-specific rather than dose-dependent response. As a cyclin-dependent kinase inhibitor regulating G1/S and G2/M checkpoints (Zhang et al., 2024), its upregulation may be linked to ionophore-induced mitochondrial dysfunction and ROS production in myocytes (Chen et al., 2014; Gao et al., 2018a; 2018b), which are known to regulate cell-cycle pathways. *COX10*, a key enzyme in mitochondrial heme A biosynthesis and cytochrome c oxidase assembly (Diaz et al., 2006), was upregulated at the highest SAL dose. This increasing expression may represent a compensatory response to maintain electron transport chain function and ATP production under mitochondrial stress. SAL induces mitochondrial dysfunction and ROS production, activating pathways such as apoptosis and autophagy (Kim et al., 2017; Klose et al., 2019; Yu et al., 2017). Even low doses of SAL can disrupt mitochondrial function (Zdanowicz et al., 2025), while higher doses exacerbate these effects, indicating a non-linear toxicity pattern and supports our results.

In contrast, the liver showed a more limited response with only significant alteration observed in *CYP21A2* gene. This gene encodes a mitochondrial CYP450 enzyme that is located on the inner mitochondrial membrane and it has a mitochondrial respiratory activity (Miller et al., 2025). SAL-induced mitochondrial dysfunction, including impaired membrane potential and increased ROS, is well studied in liver (Antonenko et al., 2022; Managò et al., 2015). Furthermore, numerous studies demonstrated that SAL alter mitochondrial gene expression in various cancer types (Cosialls et al., 2023; Kiełbasiński et al., 2020; Lu et al., 2011; Parajuli et al., 2013). Supporting these results, the downregulation of *CYP21A2* at both low and high doses suggests a non-linear, stress-related response, indicating high sensitivity to SAL exposure. In contrast, other mitochondrial-related genes (*DNM1L, MFF, MFN2*) were not significantly altered, suggesting selective modulation of mitochondrial pathways. We concluded that SAL induced tissue-specific and non-linear transcriptional responses in turkeys, with the heart showing more susceptibility compared to the liver.

Secondly, we evaluated whether MON, a commonly used polyether ionophore (Chapman, 2010), modifies SAL-induced transcriptional responses. Distinct significant transcriptional responses were observed between the SAL and MON+SAL groups in the heart. The main effect of MON co-exposure was the downregulation of SAL-induced transcriptional responses. However, MON alone did not significantly affect gene expression. Polyether ionophores such as SAL and MON share similar mode of action mechanisms (Ekinci et al., 2023). MON has been reported to affect cell-cycle progression through modulation of cyclin-dependent kinases and increased p21/p27 activity (Park et al., 2002; Park et al., 2003; Wang et al., 2018). Since both compounds target similar cellular pathways, their combined exposure may result in overlapping effects. In the present study, the lack of a significant effect of MON alone suggests that its impact may remain below the threshold required to trigger detectable transcriptional changes. However, under SAL-induced stress, MON appears to modulate these pathways.

Next, we evaluated whether ENR modifies SAL-induced transcriptional responses under co-exposure treatment. Similar to MON co-exposure, ENR altered SAL-induced responses in the heart. The main effect of ENR co-exposure was the decreasing of SAL-induced transcriptional activity. Both ionophores and fluoroquinolones have been associated with mitochondrial dysfunction and oxidative stress (Fief et al., 2019; Grabowski et al., 2024; Kim et al., 2017; Salimiaghdam et al., 2022; Yu et al., 2017). Supporting these findings, in our study, the co-exposure of ENR+SAL may lead to overlapping stress responses, and leads to modification in transcriptional responses. Unlike MON co-exposure, ENR co-exposure did not significantly affect *CDKN3*, suggesting a more limited impact on cell-cycle pathways. Although both compounds modify SAL-induced responses, their effects are pathway-specific and depend on the underlying mechanism of interaction which may be related to differences in their mode of action. In contrast, no significant changes were observed in the liver, which demonstrates that this interaction is tissue-specific and primarily affects cardiac responses. We concluded that ENR reshapes SAL-induced molecular responses in the heart, but with a more selective effect compared to MON.

To distinguish whether antibiotic and ionophore co-administration induces transcriptional changes independent of SAL exposure, MON and ENR were administered without SAL to turkeys (Supplementary Fig. 1). MON+ENR administered without SAL also altered transcriptional activity, mainly in the heart. This indicates that these compounds can independently influence pathways related to stress signalling and mitochondrial function. Some genes showed similar expression patterns to those observed with SAL. This overlap suggests that certain genes identified in this study, such as *LCN2* and *RBL1* may serve as general biomarkers of ionophore-induced cellular stress rather than being strictly SAL-specific. However, the magnitude and pattern of responses under co-exposure conditions indicate that SAL remains a major driver of transcriptional changes. The diagnostic utility of these candidate biomarkers requires further validation through correlation with functional or histopathological endpoints.

We further evaluated the effects of triple exposure (ENR+MON+SAL) on SAL-induced transcriptional responses. Compared to single and dual exposure, triple exposure caused more pronounced decrease in gene expression related to stress and mitochondria dysregulation in the heart. Importantly, gene expression was downregulated in the ENR+MON+SAL group compared to both SAL and MON+SAL groups. This may reflect the interaction of multiple stress factors. *BRCA2*, the critical regulator of genomic stability (Gorodetska et al., 2019), was also downregulated under triple co-exposure, suggesting a possible involvement of DNA damage response and repair mechanisms under more complex conditions.

In contrast to previous findings, significant changes were also observed in the liver. *CAMK2G, CREB1*, and *DNM1L* was downregulated in the ENR+MON+SAL group compared to MON+SAL. DNM1L is a regulator mitochondrial fission (Liu et al., 2021). Dysregulated mitochondria dynamics including fission have been demonstrated in drug-induced liver injury (Pi et al., 2013; Ramachandran et al., 2021). Similarly, in our study, its downregulation under the triple co-exposure suggest that mitochondria dynamics may be affected. CAMK2G is a calcium/calmodulin-dependent kinase that regulates Ca²⁺ transport (Kahl and Means, 2003). Ionophores cause increasing intracellular Ca²⁺ homeostasis, which in our case its upregulated expression in SAL and MON+SAL groups can be explained by activation Ca²⁺-dependent signalling cascades (Sandercock and Mitchell, 2004). Similarly we observed a same pattern for CREB1, a transcription factor that mediated by cAMP and calcium signaling and regulates metabolic and stress-related signals (Cui et al., 2021). The downregulation of *CAMK2G* and *CREB1* in the ENR+MON+SAL group suggests suppression of calcium and cAMP-dependent signalling pathways under combined treatment. The exposure to ENR may exceed the adaptive capacity of hepatocytes. Cells may shift from signalling activation to transcriptional suppression. Overall, these results can show that increasing interaction complexity changes the pattern of SAL-induced transcriptional responses in both tissue.

Finally, we evaluated whether TIAM modifies SAL-induced transcriptional responses. Similar to other treatments, the strongest effects were observed in the heart. In contrast to MON and ENR, TIAM showed a more pronounced effect on gene expression, downregulating stress-related genes both alone and in combination with SAL. As a CYP450 (mainly CYP3A) inhibitor, TIAM may alter ionophore biotransformation and exposure, thereby influencing metabolic and cellular responses (Szűcs et al., 2004; Witkamp et al., 1994). This may affect stress signalling and mitochondrial function, contributing to the stronger transcriptional effects observed. Despite the role of the liver in SAL metabolism, no significant transcriptional changes were detected in liver, suggesting that these interactions may occur at metabolic or post-transcriptional levels. TIAM appears to exert a stronger regulatory effect on SAL-induced responses in the heart compared to other co-exposures.

The attenuation of SAL-induced transcriptional responses under co-exposure may reflect pathway-level cross-modulation. SAL-induced expression changes may represent adaptive responses to cellular stress. Because MON, ENR, and TIAM can affect mitochondrial function, oxidative stress, and stress-related signaling pathways that are also influenced by SAL, their co-administration may cross-modulate these pathways by altering the magnitude or direction of SAL-induced signaling, resulting in downregulation of the selected genes. A comparable effect has been reported for narasin, another polyether ionophore, which attenuated TGF-β- and IL-6-induced responses by inhibiting SMAD2/3 and STAT3 signaling (Chen et al., 2020). Recent studies have also demonstrated pathway-level cross-modulation, in which drug exposure attenuated inflammatory gene expression by modulating MAPK, NF-κB, and antioxidant signaling pathways (Abdulaal et al., 2024; Izumi et al., 2025). However, because the present findings are based on mRNA expression, protein level validation of the key responsive genes would help determine the biological significance of the observed changes.

## Conclusion

This study identifies tissue-specific transcriptional responses to SAL toxicity in turkeys and demonstrates that these responses are modulated by co-administered xenobiotics. The heart showed more susceptibility than the liver, with prominent effects on mitochondrial and stress-related mechanisms. Co-exposure altered these responses, generally leading to suppression of target gene expression across treatments. Overall, these findings confirm the heart as the primary target of SAL toxicity and provide mechanistic insight into ionophore–antibiotic interactions.

## CRediT authorship contribution statement

**İlksen Berfin Ekinci:** Writing – original draft, Visualization, Methodology, Investigation, Formal analysis. **Anna Sławińska:** Writing – review & editing, Supervision, Investigation, Conceptualization. **Małgorzata Olejnik:** Writing – review & editing, Supervision, Project administration, Investigation, Funding acquisition, Conceptualization.

## Disclosures

The authors declare the following financial interests/personal relationships which may be considered as potential competing interests:

Malgorzata Olejnik reports financial support was provided by National Science Centre Poland. If there are other authors, they declare that they have no known competing financial interests or personal relationships that could have appeared to influence the work reported in this paper.

## Appendix Supplementary materials

Image, application 1

Image, application 2

Image, application 3

## Footnotes

Supplementary material associated with this article can be found, in the online version, at doi:10.1016/j.psj.2026.107509.
