# Supplementary material for: Tissue-specific molecular responses to salinomycin and their modulation by ionophore–antibiotic interactions in turkeys
Source: Poult Sci. 2026 Jul 27;105(11):107509. doi: 10.1016/j.psj.2026.107509 (PMC13476495; doi:10.1016/j.psj.2026.107509)
Supplement: Supplementary file 1 [file mmc1.docx]

**Supplementary Figure 1.**

***Monensin and enrofloxacin induce gene-specific transcriptional changes independent of salinomycin exposure***

To evaluate whether antibiotic and ionophore co-administration induces transcriptional changes independent of SAL exposure, turkeys were treated with MON (125 mg/kg), ENR (10 mg/kg b.w.), or their combination (ENR+MON), and gene expression was analyzed in the heart and liver by using RT-qPCR (Supplementary Fig. 1).

In the heart, several genes showed significant alterations between groups, including *LCN2*, *NFKBIA*, *RBL1*, and *COX10*. *LCN2* expression was downregulated in ENR+MON group (0.25 fold)compared to both control (1.0 fold) and MON-alone (1.09 fold) groups. *NFKBIA* was also downregulated in ENR+MON (0.58 fold) group compared to MON (1.28 fold). *RBL1* expression was lower in combined treatment (0.41 fold) compared to control (1.0 fold). *COX10* was downregulated in co-exposure group (0.37 fold) compared to both control (1.0 fold) and ENR-alone (1.43 fold) (Supplementary Fig. 1A). In liver only CAMK2G expression was statistically significant. Its expression was downregulated in ENR group (0.82 fold) compared to MON group (1.84 fold) (Supplementary Fig. 1B). No other pairwise comparisons were statistically significant for both tissue.

**Supplementary Fig. 1.** Transcriptional responses of target genes exposure to monensin (MON), enrofloxacin (ENR) and their combined administration (ENR+MON) in the heart (A) and the liver (B) with RT-qPCR analysis. Relative expression of each gene was calculated by the 2^−ΔΔCt^ method and normalized to the control group (=1). Statistical significance were determined using the Kruskal–Wallis test followed by Dunn’s multiple comparisons test. Data are presented as means ± SEM (n=6). The X-axis represents target genes, and the Y-axis represents the fold change of a target gene (2^−ΔΔCt^). *P < 0.05; **P < 0.01.
